# Altered Circulating Follicular T Helper Cell Subsets and Follicular T Regulatory Cells Are Indicators of a Derailed B Cell Response in Lupus, Which Could Be Modified by Targeting IL-21R

**DOI:** 10.3390/ijms232012209

**Published:** 2022-10-13

**Authors:** Krisztina Szabó, Ilona Jámbor, Kitti Pázmándi, Nikolett Nagy, Gábor Papp, Tünde Tarr

**Affiliations:** 1 Division of Clinical Immunology, Institute of Internal Medicine, Faculty of Medicine, University of Debrecen, H-4032 Debrecen, Hungary; 2 Department of Immunology, Faculty of Medicine, University of Debrecen, H-4032 Debrecen, Hungary

**Keywords:** systemic lupus erythematosus, follicular T helper cell, follicular T regulatory cell, B cell, chemokine receptors, interleukin-21 receptor

## Abstract

Systemic lupus erythematosus (SLE) is characterized by the breakdown of self-tolerance, the production of high-affinity pathogenic autoantibodies and derailed B cell responses, which indicates the importance of central players, such as follicular T helper (T_FH_) subsets and follicular T regulatory (T_FR_) cells, in the pathomechanism of the disease. In this study, we aimed to analyze the distribution of the circulating counterparts of these cells and their association with disease characteristics and B cell disproportions in SLE. We found that the increased percentage of activated circulating T_FH_ (cT_FH_) and cT_FR_ cells was more pronounced in cutaneous lupus; however, among cT_FH_ subsets, the frequency of cT_FH_17 cells was decreased in patients with lupus nephritis. Furthermore, the decreased proportion of cT_FH_17 cells was associated with low complement C4 levels and high disease activity scores. We also investigated whether the blocking of the IL-21 receptor (IL-21R) with an anti-IL-21R monoclonal antibody inhibits the B cell response, since IL-21 primarily produced by T_FH_ cells potentially promotes humoral immunity. We observed that anti-IL-21R inhibited plasmablast generation and immunoglobulin production. Our study demonstrated that, besides cT_FR_/cT_FH_ imbalance, cT_FH_17 cells play a crucial role in SLE pathogenesis, and modulating cT_FH_-B cell interaction through the IL-21/IL-21R pathway may be a promising therapeutic strategy to suppress the pathological B cell response.

## 1. Introduction

Systemic lupus erythematosus (SLE) is a prototypical chronic systemic autoimmune disease with the potential to cause damage to various organ systems, including the renal, neural, cutaneous, cardiopulmonary, musculoskeletal and hematologic systems. SLE occurs mainly in women of childbearing age. Patients with lupus frequently experience periods of remissions and exacerbations in a range of severity from mild to moderate forms that could lead to serious long-term outcomes [1,2].

It is well established in lupus that the imbalance of different subsets of B cells, the pronounced autoreactive B cell activation and antinuclear autoantibody production, as well as the subsequent formation of immune complexes (ICs), are crucial in disease pathogenesis [3]. These characteristic features are often associated with the most commonly developed manifestations that affect the skin and kidneys, such as acute cutaneous lupus erythematosus and lupus nephritis [4,5]. Indeed, in systemic autoimmune diseases, as in lupus, autoantibodies targeting general self-antigen structures are fundamental pathogenic factors, since they contribute to the development of numbers of clinical symptoms. Consequently, it is very important to explore those impairments which could lead to enhanced autoantibody production. The generation of autoantibodies is indicative of an incomplete induction of central and peripheral tolerance mechanisms, as well as B cell hyperactivity induced by altered immune regulation [6]. The first B cell defect happens in the bone marrow at the immature stage of the cells, whereas the second takes place between the transitional and mature-naive stage during their migration into lymphoid follicles. The third defect in self-tolerance relates to the microenvironment of germinal centers (GCs), where follicular dendritic cells (FDCs), follicular T regulatory (T_FR_) cells and follicular T helper (T_FH_) cells collaborate to regulate the multistage differentiation processes of antigen-specific B cell clones [7,8]. Nevertheless, the breakdown in early B cell tolerance and tightly regulated GC responses lead to the development of lupus in susceptible individuals [9]. As a result of IC deposition, ectopic or tertiary lymphoid neogenesis can be manifested within the glomeruli and tubulointerstitium in the kidney of lupus patients, which assists further in the perpetuation and escalation of autoimmunity via establishing the perfect niche for autoreactive B cell proliferation and the differentiation of autoantibody-producing cells [10,11,12,13].

Over the past decades, extensive knowledge has been accumulated on T_FH_ cells that have an important role in GC formation, where somatic hypermutation (SHM) of B cell receptor (BCR) genes, immunoglobulin (Ig) class switching and the development of memory B cells, along with high-affinity antibody-secreting plasma cells, occur [14]. These processes are regulated by T_FH_ cells, which express lineage-specific transcription factor B cell lymphoma 6 (Bcl-6) and CXC-chemokine receptor 5 (CXCR5), guiding their follicular recruitment [15,16,17]. T_FH_ cells in the follicle, and later in GCs, provide help to B cells via programmed cell death protein 1 (PD-1), CD40 ligand (CD40L, also known as CD154) and inducible co-stimulator (ICOS) cell-surface proteins [18]. Interleukin (IL)-21, a hallmark cytokine in the GC reaction is primarily produced by T_FH_ cells, and does not just support B cell differentiation via IL-21 receptor (IL-21R) signaling, but also promotes T_FH_ cell development in an autocrine fashion [19,20]. Recently, forkhead box protein 3 (FoxP3)-expressing CXCR5^+^ T_FR_ cells, a specialized subset derived from thymic regulatory T (Treg) cells, have been identified, which co-express characteristic T_FH_ and Treg markers at the same time. This cell subset is able to optimize GC B cell responses primarily by expressing cytotoxic T lymphocyte-associated protein 4 (CTLA-4) as an effector molecule [21]. Together, T_FH_ and T_FR_ cells are key regulators of the GC responses. T_FH_ cells control the size and output of the GC, while T_FR_ cells act as a fine tuner of the humoral responses [22,23]. Considering the inability to routinely measure human samples of secondary lymphoid tissue or kidney biopsy, the analysis of T_FH_-like and T_FR_-like cells in the peripheral blood have become widely prevalent. 

Circulating T_FH_ cells (cT_FH_) can be identified as CXCR5^+^ memory T cells, which are composed of functionally and phenotypically distinct subsets [24]. Of note, compared to GC T_FH_ cells in lymph nodes, cT_FH_ cells express a lower amount of canonical T_FH_ markers, but they retain their ability to effectively promote B cell response [25]. The heterogeneous population can be classified by the expression of chemokine receptors CXCR3, CCR6 and CCR7, the immunoregulatory molecule PD-1 and the co-stimulatory molecule ICOS. According to the expression of chemokine receptors, CXCR3^+^CCR6^−^ cT_FH_1-like, CXCR3^+^CCR6^+^ cT_FH_1/17-like, CXCR3^−^CCR6^−^ cT_FH_2-like and CXCR3^−^CCR6^+^ cT_FH_17-like subsets can be identified. In addition, the cT_FH_ subset mirrors its effector T cell counterparts by means of expressing specific T helper (Th) cell line transcription factors and cytokines [25]. Moreover, cT_FH_ cells can also be divided into activated and quiescent subsets based on CCR7, ICOS and PD-1 expression, with ICOS^+^PD-1^++^CCR7^lo^ resembling activated memory cT_FH_ cells, while ICOS^−^PD-1^+^CCR7^int^ and ICOS^−^PD-1^−^CCR7^hi^ correspond to quiescent memory cT_FH_ cells [26]. In terms of their functional role and genetic profile, ICOS^+^PD-1^++^CCR7^lo^ or ICOS^−^PD-1^+^CCR7^int^ phenotypes within cT_FH_2 and cT_FH_17 cells are efficient at supporting naive B cells to differentiate into isotype-switched Ig-producing plasma cells. By contrast, T_FH_1 cells lack the ability to aid naive B cells; however, they can provide help to memory B cells if they are in an activated state [27,28]. 

Similar to cT_FH_ cells, an increasing number of studies discovered a circulating counterpart of lymphoid tissue T_FR_ cells at the periphery, termed cT_FR_ cells. Regarding their origin, cT_FR_ cells derive from pre-GC T_FR_ cells, exiting secondary lymphoid tissue prior to encountering B cells and completing their full differentiation. Thus, human cT_FR_ cells express Bcl-6, PD-1 and ICOS at a lower level and retain the expression of IL-2R alpha chain (CD25), while mature T_FR_ cells in GCs progressively lose CD25 expression as they approach the end of their differentiation process [22,29,30]. 

Increased cT_FH_ cell ratios and absolute cell numbers have been reported in SLE over the past years. The association between cT_FH_ hyperactivity, disease activity scores and the presence of antinuclear autoantibodies revealed the prominent role of T_FH_ cells in lupus pathogenesis [31]. With their sophisticated molecular network in GCs, T_FH_ cells can serve as an important therapeutic target and provide a novel way to modulate pathogenic autoantibody production. So far, cT_FH_ phenotypic plasticity and the heterogeneity of the disease, medication and cohort size have generated controversial results in SLE [32,33,34,35]. Thus, in this study, we aimed to clarify the imbalance of cT_FH_ cell subsets and cT_FR_ cells, as well as determine their relationship with disease characteristics and B cell disproportions in patients with lupus.

## 2. Results

### 2.1. Assessment of the Frequency of Activated cT_FH_ and cT_FR_ Cells in Lupus Patients and Healthy Individuals

**Figure 1:**
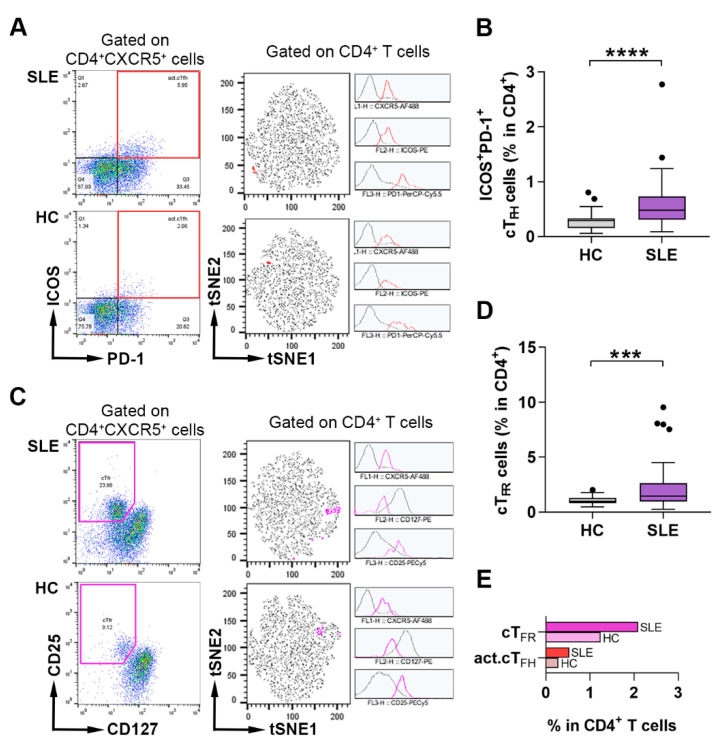
The distribution of activated cT_FH_ and T_FR_ cells in lupus patients and healthy individuals. Peripheral blood mononuclear cells (PBMCs) were isolated from 48 SLE patients and 36 healthy controls (HC) were then stained with fluorochrome-conjugated monoclonal antibodies, as described in Materials and Methods. The proportions of peripheral activated (act.) cT_FH_ cells and cT_FR_ cells were quantified as their percentage within CD4^+^ lymphocytes. (**A**) Representative dot plots from an active lupus patient and HC show the identification of activated cT_FH_ cells using ICOS and PD-1. Merged tSNE plots incorporating 30,000 CD4^+^ T cells from the corresponding lupus patient and healthy control show the act.cT_FH_ cluster identified by tSNE clustering analysis (red color represents act.cT_FH_). Histograms show the expression of markers used to identify act.cT_FH_ cells. (**B**) Percentages of act.cT_FH_ cells. (**C**) Representative dot plots from an active SLE patient and HC show the identification of cT_FR_ cells by CD127 and CD25. Merged tSNE plots incorporating 30,000 CD4^+^ T cells from the corresponding lupus patient and healthy control show the cT_FR_ cluster identified by t-SNE clustering analysis (magenta color represents cT_FR_). Histograms show the expression of markers used to identify cT_FR_ cells. (**D**) Percentages of cT_FR_ cells. (**E**) The proportions of act.cT_FH_ and cT_FR_ clusters among CD4^+^ T cells were indicated by the horizontal bars. Mann–Whitney nonparametric test was used (**C**,**E**). Box plots represent the interquartile range (IQR) with a line in the middle as the median. Statistically significant differences are indicated by *** *p* < 0.001; **** *p* < 0.0001.

We investigated the percentages of activated cT_FH_ and cT_FR_ cells in the peripheral blood of SLE patients and healthy controls. Activated cT_FH_ cells were identified as ICOS^+^PD-1^+^ cells, while CD25^+^CD127^−/lo^ cells were determined as cT_FR_ cells among CD4^+^CXCR5^+^ T cells, then their frequencies were quantified within CD4^+^ lymphocytes. A nonlinear dimensionality reduction visualization tool, namely, t-distributed stochastic neighborhood embedding (tSNE), was used to visualize the relative proportion of each cell subset in CD4^+^ T cell populations in a representative patient and control sample (Figure 1A,C). As previously described, cT_FH_ cells belong to the CD45RA^−^ memory T cell population, thus we evaluated the percentages of CD45RA^+^ naive and CD45RA^−^ memory T cells among circulating CD4^+^CXCR5^+^ and CD4^+^CXCR5^−^ T cells. We found that the ratio of memory phenotypes increased two-fold (78.83 ± 4.61% vs. 36.48 ± 6.43%; *p* < 0.0001), while the ratio naive cells decreased three-fold in CXCR5^+^ Th cells compared to CXCR5^−^ T cells (20.63 ± 4.67% vs. 63.05 ± 6.49%; *p* < 0.0001) (Supplementary Figure S1). We previously reported that lupus patients had elevated frequency of activated cT_FH_ cells, which was more pronounced in patients with anti-dsDNA positivity compared to controls [16]. To endorse and refine our analysis, besides activated cT_FH_ cells, other T_FH_ subsets were further characterized in the peripheral blood of lupus patients. Consistently, the percentages of activated cT_FH_ cells were also increased significantly in patients with SLE in comparison to healthy controls (0.58 ± 0.44% vs. 0.29 ± 0.16%; *p* < 0.0001) (Figure 1B). When patients were divided into subgroups according to the SLE Disease Activity Index (SLEDAI) and the presence of anti-dsDNA, the percentages of activated cT_FH_ cells were elevated further in case of patients who had abnormal levels of serum anti-dsDNA compared to controls (0.58 ± 0.31% vs. 0.29 ± 0.16%; *p* = 0.0005); however, it was less obvious in patients with a higher SLEDAI (Supplementary Figure S2A). Contrary to previous conflicting reports [34,35], the frequencies of cT_FR_ cells were significantly higher in patients with lupus than in controls (2.26 ± 2.09% vs. 1.06 ± 0.36%; *p* = 0.0003) (Figure 1D) and, interestingly, it was further elevated in patients with a lower SLEDAI and autoantibody values (Supplementary Figure S2B).

### 2.2. The Imbalance of the Proportions of cT_FH_ Cell Subsets and Their Correlation with Disease Phenotype in SLE Patients

**Figure 2:**
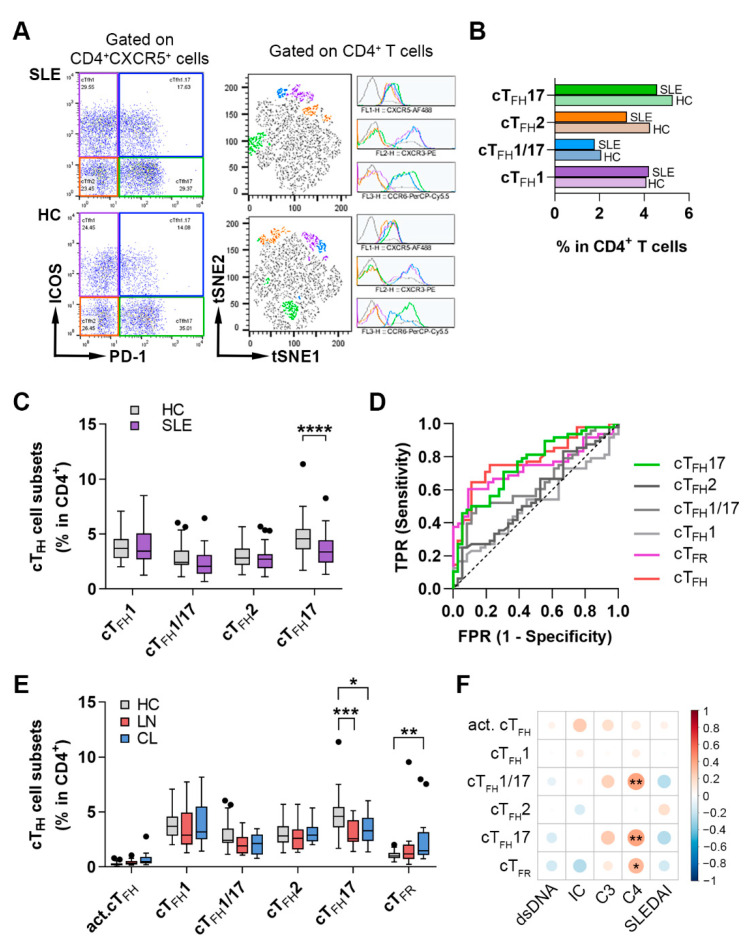
The imbalance of cT_FH_ subsets in SLE patients and their association with disease characteristics. PBMCs were isolated from 48 SLE patients and 36 healthy controls (HC) were then stained with fluorochrome-conjugated monoclonal antibodies, as described in Materials and Methods. The percentages of blood cT_FH_ cell subsets were quantified as their percentage within CD4^+^ lymphocytes. (**A**) Representative dot plots from an active SLE patient and HC show the identification of cT_FH_ subsets: cT_FH_1 (CXCR3^+^CCR6^−^), cT_FH_1/17 (CXCR3^+^CCR6^+^), cT_FH_2 (CXCR3^−^CCR6^−^) and cT_FH_17 (CXCR3^−^CCR6^+^) cells. Merged t-SNE plots incorporating 30,000 CD4^+^ T cells from the corresponding lupus patient and healthy control show cT_FH_ clusters identified by t-SNE clustering analysis (each color represents a different cluster: cT_FH_1, purple; cT_FH_1/17, blue; cT_FH_2, orange; cT_FH_17, green). Histograms show the expression of markers used to identify each cT_FH_ subset. (**B**) The proportions of cT_FH_ clusters among CD4^+^ T cells were indicated by the horizontal bars. (**C**) Percentages of cT_FH_ subsets. (**D**) The receiver operating characteristic (ROC) curves were performed to evaluate the efficiency of cT_FH_ cell subsets to predict lupus when compared to controls. Colored lines indicate areas under the ROC curves (AUC) which are considered acceptable. (**E**) Percentages of different cT_FH_ subsets in patients with lupus nephritis (LN, *n* = 15), cutaneous lupus (CL, *n* = 13) and HC. (**F**) Correlation matrix represents the association between the percentages of cT_FH_ cell subsets, cT_FR_ cells and different serological parameters (anti [α]-dsDNA, immune complex [IC], complement [C] 3, 4 and SLEDAI) in patients with SLE. Positive correlations are displayed in red and negative correlations in blue. Color intensity and the size of the circle are proportional to the Spearman’s correlation coefficient. Two-way analysis of variance (ANOVA) with a Sidak post hoc test was used. Box plots represent the interquartile range (IQR) with a line in the middle as the median. Statistically significant differences are indicated by * *p* < 0.05; ** *p* < 0.01; *** *p* < 0.001; **** *p* < 0.0001.

Based on the different expression profiles of CXCR3 and CCR6 chemokine receptors, cT_FH_ cells were divided into four subsets, including Th1-like cT_FH_1 (CXCR3^+^CCR6^−^), cT_FH_1/17 (CXCR3^+^CCR6^+^), Th2-like cT_FH_2 (CXCR3^−^CCR6^−^) and Th17-like cT_FH_17 (CXCR3^−^CCR6^+^) cells. The percentages of these cell subsets were quantified within the CD4^+^ lymphocyte population. We applied tSNE to identify all cell subsets that expressed CXCR3 and/or CCR6, besides CXCR5, and representative samples were visualized (Figure 2A). The proportions of each lymphocyte subset among CD4^+^ T cells were indicated by the horizontal bars (Figure 2B). There was no detectable difference in the percentages of cT_FH_1, cT_FH_1/17 and cT_FH_2 subsets in lupus patients compared to controls, except for the frequencies of cT_FH_17 cells that decreased significantly in SLE compared to control values (3.46 ± 1.41% vs. 4.90 ± 1.78%; *p* < 0.0001) (Figure 2C). We did not detect substantial differences regarding the presence of autoantibodies; however, cT_FH_17 percentages tended to decrease in patients with active disease compared to controls (Supplementary Figure S2C). In addition, logistic regression and receiver operating characteristic (ROC) curve analyses were performed to explore the efficiency of cT_FH_ cell subsets to predict lupus when compared to controls. The areas under the ROC curves (AUC) 0.7 to 0.8 are considered acceptable or fair, whereas values below 0.6 mean that there is no apparent difference between the values of the two groups. The AUC for activated cT_FH_, cT_FR_ and cT_FH_17 cells were 0.7763 (95% confidence interval [CI] 0.6761 to 0.8766; *p* < 0.0001), 0.7323 (95% CI 0.6235 to 0.8412; *p* = 0.0004) and 0.7546 (95% CI 0.6515 to 0.8578; *p* < 0.0001), respectively (Figure 2D). 

Furthermore, we analyzed the percentages of cT_FH_ subsets and cT_FR_ cells in peripheral blood of patients with lupus nephritis (LN, *n* = 15) and cutaneous lupus (CL, *n* = 13), as well. Interestingly, the proportions of cT_FR_ cells were significantly higher only in CL in contrast to controls (2.61 ± 2.43% vs. 1.06 ± 0.36%; *p* = 0.0061), while the percentages of cT_FH_17 cells were diminished more markedly in LN compared to controls (3.11 ± 1.18% vs. 4.90 ± 1.78%; *p* = 0.0002) than in CL (3.49 ± 1.41% vs. 4.90 ± 1.78%; *p* = 0.0177) (Figure 2E). Although the frequencies of activated cT_FH_ cells showed a more obvious elevation in CL patients, the difference was not significant (Figure 2E).

The correlation analyses revealed that the percentages of cT_FH_1/17 (R = 0.4078; *p* = 0.0040), cT_FH_17 (R = 0.4083; *p* = 0.0040) and cT_FR_ (R = 0.3349; *p* = 0.0200) showed a significant positive correlation with serum complement C4 titer (Figure 2F). The frequencies of activated cT_FH_ cells were positively associated, while the percentages of cT_FR_ cells were negatively associated with the serum level of ICs, but no statistically significant difference was found (Figure 2F).

### 2.3. Changes in the Distribution of Circulating B Cell Subsets Is a Distinctive Characteristic of SLE

**Figure 3:**
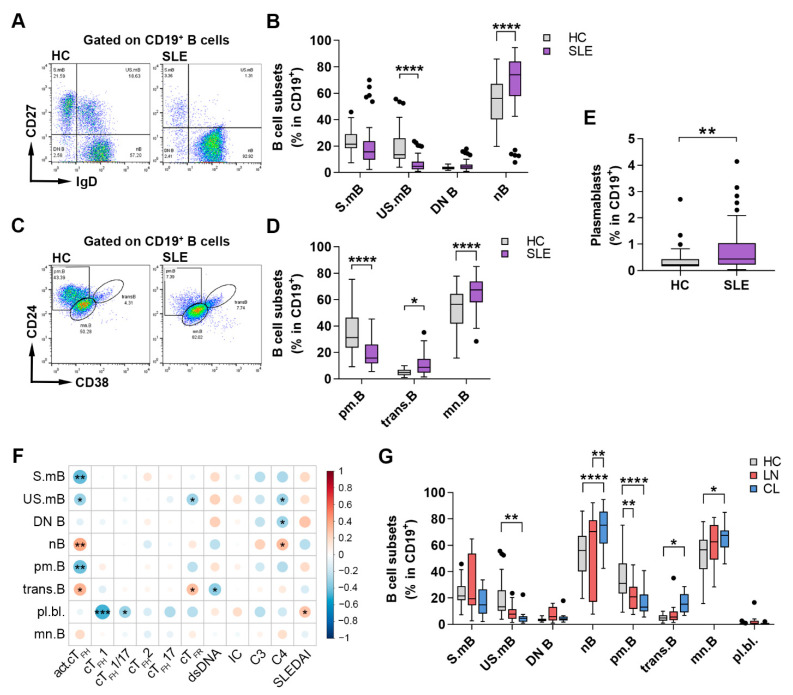
Abnormal B cell distribution and their association with SLE laboratory and clinical features. PBMCs were isolated from 48 SLE patients and 36 healthy controls (HC), and were then stained with fluorochrome-conjugated monoclonal antibodies, as described in Materials and Methods. The percentages of B cell subsets were quantified as their percentages within CD19^+^ lymphocytes. (**A**) Representative dot plots show the determination of IgD^+^CD27^−^ naive B (nB) cells, IgD^+^CD27^+^ un-switched memory B (US.mB) cells, IgD^−^CD27^+^ switched memory B (S.mB) cells and IgD^−^CD27^−^ double negative (DN) B cells. (**B**) The proportions of naive and memory B cell subsets. (**C**) Representative dot plots show the identification of CD38^hi^CD24^hi^CD27^−^ transitional B (trans.B) cells, CD38^int^CD24^int^ mature-naive B (mn.B) cells and CD38^−^CD24^hi^CD27^+^ primarily memory B (pm.B) cells. (**D**) The proportions of trans.B, mn.B and pm.B cell subpopulations. (**E**) The percentages of CD38^+^CD27^hi^ plasmablasts. (**F**) Correlation matrix represents the association between the percentages of B cell subsets and cT_FR_ cells, cT_FH_ subpopulations, as well as routine laboratory parameters (anti [α]-dsDNA, immune complex [IC], complement [C] 3, 4 and SLEDAI) in patients with SLE. Positive correlations are displayed in red and negative correlations in blue. Color intensity and the size of the circle are proportional to the Spearman’s correlation coefficient. (**G**) Percentages of different B cell subsets in patients with lupus nephritis (LN, *n* = 15), cutaneous lupus (CL, *n* = 13) and HC. Two-way analysis of variance (ANOVA) with a Sidak post hoc test was used. Box plots represent the interquartile range (IQR) with a line in the middle as the median. Statistically significant differences are indicated by * *p* < 0.05; ** *p* < 0.01; *** *p* < 0.001; **** *p* < 0.0001.

B cell immunophenotyping is a promising tool for evaluating abnormal B cell activation in lupus patients. In parallel with analyzing the distribution of cT_FH_ subsets, we also assessed the distribution of B cell subsets in order to explore the T cell-dependent B cell response in SLE pathogenesis. Memory B cell subsets and naive or mature-naive B cells were identified according to IgD, CD27, CD38 and CD24 expression as follows: IgD^+^CD27^−^ naive B cell, IgD^+^CD27^+^ un-switched memory B cell, IgD^−^CD27^+^ switched memory B cell, IgD^−^CD27^−^ double negative (DN) B cell, CD38^hi^CD24^hi^CD27^−^ transitional B cell, CD38^int^CD24^int^ mature-naive B cell, CD38^−^CD24^hi^CD27^+^ primarily memory B cell and CD38^+^CD27^hi^ plasmablasts among CD19^+^ B lymphocytes (Figure 2A,C). The percentages of both naive B cells (67.93 ± 22.93% vs. 53.20 ± 16.88%; *p* < 0.0001) (Figure 3B) and mature–naive B cells (65.13 ± 11.51% vs. 51.97 ± 15.66%; *p* < 0.0001) (Figure 3D) were significantly elevated in lupus patients compared to healthy controls. Furthermore, we found a significant increase in the percentages of transitional B cells in SLE patients compared to healthy participants (11.15 ± 8.08% vs. 5.03 ± 2.51%; *p* = 0.0487). In contrast to early and naive B cell phenotypes, the proportions of un-switched memory B cells (6.74 ± 6.02% vs. 19.41 ± 13.50%; *p* < 0.0001) (Figure 3B), as well as primarily memory B cells (18.87 ± 9.99% vs. 36.90 ± 15.99%; *p* < 0.0001) (Figure 3D), were significantly diminished in SLE patients compared to controls. On the other hand, patients with SLE had a significantly increased frequency of plasmablasts compared to control subjects (0.79 ± 0.89 vs. 0.38 ± 0.49%; *p* = 0.0056) (Figure 3E). 

By evaluating the relationship between different B cell subsets and the serological disease characteristics of SLE, we observed a significant positive correlation between the serum levels of complement C4 and the frequencies of naive B cells (R = 0.310; *p* = 0.0318) (Figure 3F). The SLEDAI score was positively associated with plasmablast frequencies (R = 0.288; *p* = 0.047) and DN B cell percentages (R = 0.284; *p* = 0.051), but in the case of the latter, no statistically significant difference was found (Figure 3F). In addition, a significant negative correlation was observed between the serum level of anti-dsDNA and the proportions of transitional B cells (R = −0.3452; *p* = 0.0175) (Figure 3F). Upon assessing the possible associations between different B cell and cT_FH_ subsets, we found a significant positive correlation between activated cT_FH_ cells and naive B cells (R = 0.3834; *p* = 0.0071) and transitional B cells (R = 0.3409; *p* = 0.0177). On the contrary, cT_FH_ cells showed significant negative correlations with switched memory B cells (R = -0.4347; *p* = 0.0020), un-switched memory B cells (R = −0.3117; *p* = 0.0310) and primarily memory B cells (R = −0.4137; *p* = 0.0035) (Figure 3F). The percentages of plasmablasts correlated negatively with the percentages of both cT_FH_1 (R = −0.4895; *p* = 0.0004) and cT_FH_1/17 cells (R = −0.3104; *p* = 0.0318) (Figure 3F). Interestingly, the percentages of cT_FR_ cells were positively associated with the proportions of transitional B cells (R = 0.3002; *p* =0.0382), while they were negatively correlated with the frequencies of un-switched memory B cells (R = −0.3046; *p* = 0.0353) (Figure 3F).

Similar to cT_FH_ cell subpopulations, the distribution of B cell subsets was also measured in patients with LN and CL in comparison with controls. We found that lupus patients with CL had more greatly decreased percentages of un-switched memory B cells (5.31 ± 5.55% vs. 19.41 ± 13.50%; *p* = 0.0071), primarily memory B cells (16.20 ± 9.55% vs. 36.90 ± 15.99%; *p* < 0.0001) and more greatly heightened proportions of naive B cells (72.84 ± 16.86% vs. 53.20 ± 16.88%; *p* < 0.0001) and mature-naive B cells (65.28 ± 9.57% vs. 51.97 ± 15.66%; *p* = 0.0151), as well as transitional B cells (16.34 ± 7.36% vs. 5.03 ± 2.51%; *p* = 0.0472), than patients with LN compared to controls (Figure 3G). Interestingly, in case of naive B cell percentages, patients with CL had significantly elevated values than patients with LN (72.84 ± 16.86% vs. 55.49 ± 29.08%; *p* = 0.0035) (Figure 3G).

### 2.4. Anti-IL-21R Inhibits the T_FH_ Cell-Dependent B Cell Differentiation in SLE Patients and Healthy Individuals

**Figure 4:**
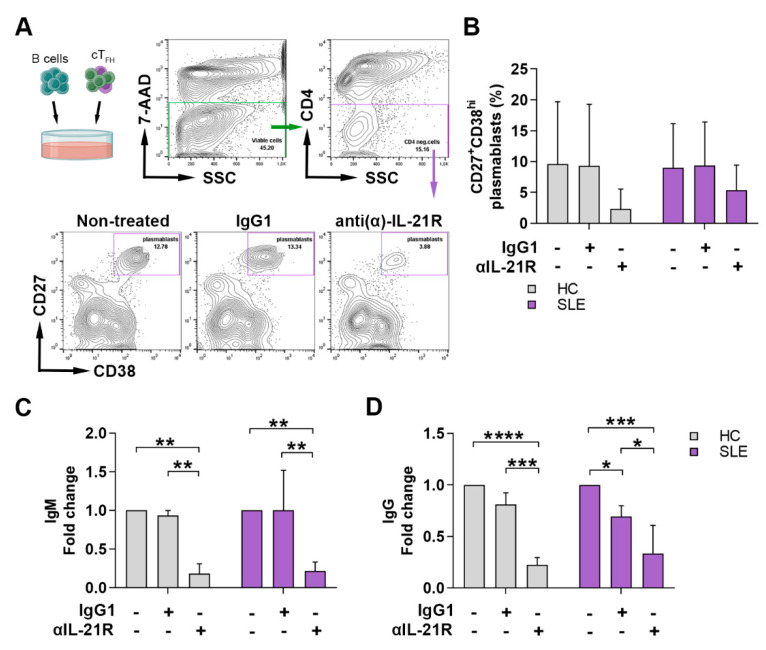
Anti (α)-IL-21R inhibits T_FH_ cell-dependent B cell differentiation in SLE patients and healthy individuals. (**A**) Representative contour plots show the gating strategy and the blocking of plasmablast differentiation by αIL-21R in SLE. As an isotype control, the IgG1 protein was used. (**B**) Percentages of 7-AAD^−^CD4^−^CD27^+^CD38^hi^ viable plasmablasts were measured by flow cytometry in healthy controls (HC; *n* = 3) and in SLE patients (*n* = 3). Fold change of IgM (**C**) and IgG (**D**) was measured by ELISA in patients with lupus and HC. Bar charts represent mean ± SD. Data analysis was performed with two-way ANOVA followed by Tukey’s multiple comparison tests. Statistically significant differences are defined as * *p* < 0.05; ** *p* < 0.01; *** *p* < 0.001; **** *p* < 0.0001.

It is widely known that T_FH_ cells play a crucial role in providing help to B cells via a balanced expression of co-stimulatory molecules and cytokines, including IL-21. Our previous work indicated that the elevated expression of IL-21^+^ cT_FH_ cells was positively associated with SLE disease activity, the presence of anti-dsDNA autoantibodies, the level of serum ICs, as well as the proportions of plasmablasts [16]. Furthermore, both cT_FH_ and B cells express IL-21R, and their differentiation and function depend on the signaling through this pathway. To determine whether blocking IL-21R with the anti-IL-21R monoclonal antibody (αIL-21R) inhibits the B cell response, we co-cultured purified B cells with autologous cT_FH_ cells in the presence of staphylococcal enterotoxin B (SEB) superantigen in vitro and investigated the ability of B cells to differentiate into CD27^+^CD38^hi^ plasmablasts and to produce IgM and IgG. The distribution of cT_FH_ and B cell subsets in PBMCs from patients and controls, and the clinical features of patients with SLE subjected to αIL-21R treatment, are detailed in Supplementary Figure S3 and Table S1, respectively. Blockade of IL-21R reduced the generation of plasmablasts both in lupus and controls, but no statistically significant difference was found (Figure 4A,B). We measured the fold change of IgG and IgM production and found that the inhibition of IL-21R decreased the production of both IgM (SLE, non-treated vs. αIL-21R: *p* = 0.0028; IgG1 vs. αIL-21R: *p* = 0.0027) and IgG (SLE, non-treated vs. αIL-21R: *p* = 0.0001; IgG1 vs. αIL-21R: *p* = 0.0148; non-treated vs. IgG1: *p* = 0.0353) in lupus similarly to controls (Figure 4C,D). Taken together, these data suggest that αIL-21R treatment can modify T_FH_-dependent B cell differentiation and function.

## 3. Discussion

Pathologic autoantibody production and its use as a diagnostic tool is the hallmark of autoimmune diseases. Still, far too little is known regarding the complex cellular and molecular mechanisms regulating antibody and autoantibody formation. Recently, multiple investigations into the role of T_FH_ cells and T_FR_ cells in coordinating B cell responses have emerged indicating that they are instrumental in promoting autoimmunity as well. T_FH_ cells are acknowledged as a distinct subpopulation of CD4^+^ Th cells that initiates and maintains extrafollicular and GC responses by mediating B cell survival, proliferation, selection and differentiation, whereas T_FR_ cells modify the outcome of GCs through ensuring a stronger competition among B cells to access T_FH_-derived help [36]. Considering their localization and the easier acquisition of lymphoid tissues in animal models, these mechanisms have been primarily studied in murine models, while accessing human lymphoid tissue samples remains challenging. Therefore, most studies in humans have focused on peripheral blood T_FH_ and T_FR_ cells and revealed that they are the memory counterparts of the lymphoid originals [37,38]. 

There are multiple human studies demonstrating increased frequencies of cT_FH_ cells in SLE patients, especially in those with moderate to severe disease activity, and their association with anti-dsDNA production [31,39,40,41,42,43]. Certain differences in the results of previous investigations can be observed due to the different methods of cT_FH_ cell identification. Since the determination of these cells is usually performed with different combinations of cell-surface markers, they could be defined as ICOS^+^ [31,42], PD-1^+^ [40,41] or ICOS^+^PD-1^+^ [34,43] cells within CD4^+^CXCR5^+^ T lymphocytes. Nevertheless, despite the variety of labeling methods, their relevance in lupus pathogenesis is obvious. Our previous work also showed that lupus patients with positive anti-dsDNA antibody titer and higher disease activity (SLEDAI score ≥ 6) have elevated percentages of IL-21-producing cT_FH_ cells. The frequencies of activated cT_FH_ cells were heightened only in patients with the presence of autoantibodies compared to healthy controls, but it was not associated with disease activity [44]. Our present results concerning the distribution of ICOS^+^PD-1^+^-activated cT_FH_ cells are consistent with findings from previous studies. Precisely, increased percentages of cT_FH_ cells were found especially in patients with moderate disease intensity and autoantibody presence compared to healthy controls. In spite of the slight elevation in cT_FH_ cell proportions in these subgroups, a positive correlation between the frequencies of activated cT_FH_ cells and the SLEDAI or anti-dsDNA have not been found in our cohort. We observed a marked positive association between activated cT_FH_ cells and the serum titer of ICs; however, it was not significant. Although other authors reported a positive correlation between cT_FH_ cells and disease severity, these diverse results could merely reflect the differences between the study cohorts. In a recent study, Sasaki et al. performed immune cell profiling using longitudinal mass cytometry analysis in peripheral blood mononuclear cells (PBMCs) from patients with early and with established SLE. The analysis revealed that cT_FH_ cells were expanded in the early phase of lupus and decreased longitudinally, while a recently described cell type, namely, peripheral T helper cells (T_PH_), remained elevated during the first year post-enrollment. Their hypothesis was that T_FH_-B cell interactions in lymphoid tissues may be important at the onset of the disease, which may then be changed to T_PH_-B cell interplay at the site of inflammation in time [45]. Interestingly, when they measured the correlation between serum chemokine levels and immune cells, CCL20 was strongly correlated with expanded cT_FH_ cells. CCL20 is an inflammatory mediator whose expression was detectable during renal inflammation in an experimental murine model [46]; thus, cT_FH_ cells may migrate toward the CCL20 gradient. In our study, we observed that percentages of activated cT_FH_ cells also decreased with the duration of the disease (Supplementary Figure S2D,E).

The recently described T_FR_ cells which play a role in modulating T_FH_ function and maintaining the balance of the GC reaction seem to be a significant factor in SLE pathogenesis. So far, controversial results have been published; both decreased [34,47] and increased [35] frequencies of cT_FR_ cells were reported in SLE. This discrepancy may arise from the difference in phenotypic analyses and the composition of study cohorts. The peripheral counterpart of T_FR_ cells is detectable in the peripheral blood as CD25^+^CD127^−/lo^ or Foxp3^+^ cells within CD4^+^CXCR5^+^ T lymphocytes. Actually, in certain studies, they were further divided into phenotypically and functionally distinct subpopulations, including CD45RA^+^FoxP3^+^ resting, CD45RA^−^FoxP3^high^ activated and CD45RA^–^FoxP3^+^ non-suppressive cT_FR_ cells. It was also discovered that the percentages of activated cT_FR_ cells were decreased, whereas non-suppressive cT_FR_ subsets were elevated in lupus patients compared to healthy controls [47,48]. In our investigation, we found that CD25^+^CD127^−/lo^ cT_FR_ cells were increased in SLE patients compared to controls; of note, the difference was more pronounced in patients with inactive disease/mild disease activity and fewer autoantibody titers. Considering that CD25^+^CD127^−/lo^ cells are more likely to express FoxP3 at moderate to high level [49], in our experiment, cT_FR_ cells may have represented both activated and non-suppressive subtypes. Since circulating CXCR5^+^ T cells expressed a low amount of CD45RA and non-suppressive cT_FR_ represented a considerable part against the activated subtype within the CD45RA^−^ population, the results of our study are consistent with these findings. We also evaluated the relationship between cT_FR_ cell distribution and clinical features and found that the frequencies of cT_FR_ cells were higher in patients with cutaneous lesions than in patients with nephritis. According to Fonseca et al., blood T_FR_ cells are generated and exit the lymphoid tissue before reaching complete differentiation towards genuine T_FR_ cells, which could affect their ability to fully suppress humoral responses [29]. Interestingly, fully matured tissue-resident T_FR_ cells have low CD25 expression, while presenting a T_FH_-like gene expression profile, which suggests a negative impact of IL-2 on their late differentiation. On the other hand, blood T_FR_ cells express CD25, and in their immature state they closely resemble natural Treg cells, but their humoral suppression capacity is less effective [29,47]. Additionally, a study reported the hypermethylation of conserved non-coding sequence (CNS) 2 element at the FoxP3 locus in lupus cT_FR_ cells, which could explain the functional decline examined in SLE [48]. A balanced IL-2/CD25 signaling is critical for maintaining not just T_FR_ but also T_FH_ generation, because IL-2 could suppress both cells; nevertheless, it is required for Treg development. Two studies demonstrated that a carefully adjusted, prolonged low-dose IL-2 treatment led to the recovery of T_FR_/T_FH_ immune balance and successfully mitigated pathogenic B cell responses, as well as kidney damage [50,51]. All these results support the use of T_FR_ cells as an attractive therapeutic target in lupus.

It has recently been shown that human cT_FH_ cells consist of phenotypically and functionally distinct subsets. So far, controversial results have been demonstrated concerning the distribution of cT_FH_ subsets in SLE. One study reported no imbalance among cT_FH_ subsets [32], whereas another showed that the proportions of cT_FH_2 and cT_FH_17 cells were increased, while the cT_FH_1 cell ratio was decreased in lupus patients [33]. In another study, elevated percentages of cT_FH_17 were found, which was not associated with disease features. Interestingly, the frequency of these cells was further elevated after methylprednisolone treatment [52]. In addition, Yang et al. found a diminished cT_FH_1 ratio in lupus patients, which was negatively associated with SLEDAI scores, suggesting its contribution to disease pathogenesis. Due to the high expression of CXCR3^+^ T cells in lupus skin samples, it was implied that cT_FH_1 cells may have migrated into the skin [53]. Our results demonstrate that only the proportions of the cT_FH_17 cell subset differed significantly in SLE compared to controls, and the decrease was more striking in patients with active disease/moderate disease activity. Furthermore, when patients were grouped according to their clinical symptoms, cT_FH_17 proportions were greatly reduced in lupus patients with nephritis compared to controls, while the difference was less apparent in patients with cutaneous lesions. Taking into consideration that decreased cT_FH_17 percentages were associated with low titers of complement C4 protein and high SLEDAI scores, according to an explanation similar to Yang’s [53], cT_FH_17 cells may migrate into the site of inflammation, especially into the kidney, rather than remain in the circulation. Numerous studies have established the pathogenic role of the Th17/IL-17 axis in lupus, with a special emphasis in lupus nephritis [54]. Blood T_FH_17-like cells seem to share many features with genuine Th17 cells, including their IL-17 and IL-21 cytokine, RORγt transcription factor, as well as CCR6 chemokine receptor expression. There are several investigations that observed the recruitment of circulating CCR6^+^ Th17 or cT_FH_ cells in response to chemokine ligand production in kidney tissues [55,56,57,58,59]. In a murine lupus model, the inhibition of a multifunctional serine/threonine kinase, namely, calcium/calmodulin-dependent protein kinase IV (CaMK4), led to the decrease in CCR6 and CCL20 expression, as well as to the amelioration of glomerular injury, which highlighted the role of the CCR6/CCL20 axis in SLE [60].

Besides cT_FH_ cell subsets, we assessed a wide variety of B cell subpopulations to explore the relationship between cT_FH_ and B cells in lupus, as well. The altered distribution of B cell subsets had been already confirmed in our previous investigation [44], and the present cohort showed a similar pattern to others [61,62]. Briefly, the percentages of pre-GC B cells, namely, transitional and naive B cells, were increased, while the frequencies of primarily and un-switched memory B cells were decreased in lupus compared to controls. Consistent with other observations, we also detected heightened plasmablast frequencies in patients with lupus compared to controls, and it correlated positively with disease activity [63]. Interestingly, when we assessed B cell distributions according to skin or kidney involvement, the established differences were more definite in patients with cutaneous lesions than nephritis. On the other hand, proportions of both plasmablast and DN B cells were markedly elevated in patients with kidney damage rather than patients with skin lesions. Of note, the percentages of DN B cells associated negatively with serum titers of complement C4 protein. Characterization of distinct B cell populations revealed some under-represented cell types, including DN B cells, which mainly consist of primed antibody-secreting cell precursors arisen through the extrafollicular differentiation pathway [64]. The increased ratio of these cells in SLE was associated with higher SLEDAI scores and an anamnestic history of kidney injury [65,66,67]. Corresponding results were found in our study, as the percentages of DN B cells increased to a greater degree in patients with nephritis, compared to patients with cutaneous lupus, although the difference was not statistically significant. Correlation analysis revealed a significant negative correlation with complement C4 titers and a positive, but not significant, association with disease activity. Hence, according to our data, activated cT_FH_ cells showed a positive correlation with pre-GC B cell subsets, such as transitional and naive B cells, but were negatively associated with memory B cell subpopulations, indicating an ongoing GC response in secondary lymphoid tissue. The positive association between cT_FR_ cells and transitional B cells, as well as their negative correlation with un-switched memory B cells, may also suggest a proceeding humoral immune response. Another possible explanation may be that high levels of IL-21 in lymphoid tissues restrict T_FR_ differentiation via the reduction of IL-2Rα and FoxP3; thus, the increased generation of immature T_FR_ cells and their exit into the circulation could be a compensatory mechanism [68,69]. Further correlation analyses shed light on the negative association of the percentages of plasmablasts and cT_FH_1, cT_FH_1/17 and cT_FH_17 cells. Although the association with cT_FH_17 cells was not significant, the trend was consistent with previous observations and could indicate a cT_FH_17-induced plasmablast differentiation in affected tissues [70]. In addition, ROC curve analysis of activated cT_FH_ and cT_FH_17 cells suggested that they could serve as biomarkers with moderate accuracy to distinguish lupus from healthy individuals.

IL-21 is the hallmark cytokine of T_FH_ cells, which acts directly on B cells through the IL-21R/TET2/AIM2/c-MAF pathway to regulate the differentiation and function of not only B cells, but T_FH_ cell polarization as well. In an MRL-Fas(lpr) lupus murine model, blocking the IL-21 signal with an IL-21R.Fc fusion protein reduced the phenotypic disease severity, such as proteinuria, skin lesions, as well as renal injury and autoantibody production [71,72]. Investigations in autoimmune BXD2 mice demonstrated that IL-21 promoted T_FH_ and Th17 cell differentiation, as well as GC formation, while it suppressed T_FR_ development and function [68]. Another study with lupus-prone B6.*Sle1.Yaa* mice showed that treatment with an anti-IL-21 blocking antibody reduced titers of autoantibodies, decreased renal infiltrating T_FH_ cells and delayed the progression of glomerulonephritis [73]. In humans, a study investigated the importance of IL-21 in SLE and found that CD4^+^ T cells producing IL-21 were elevated in patients with lupus, and it associated positively with the ratio of Th17 cells [74]. In the present study, we evaluated the ability of αIL-21R to restrain B cell differentiation and function and found that the generation of CD27^+^CD38^hi^ plasmablasts was reduced in the cT_FH_–B cell co-culture gained from lupus patients and control individuals. The blockade of IL-21R decreased the production of both IgM and IgG; however, the difference was more striking in the case of IgG. Interestingly, both IgM and IgG decreased more in the patient with polyarthritis than in patients with a skin rash among the three patients with SLE. Further examination of B cell differentiation in response to IL-21/IL-21R inhibition in a larger cohort of lupus patients with different clinical manifestations would be beneficial in the future. Taken together, the modulation of T_FH_–B cell interaction using αIL-21R may be an attractive therapeutic approach to halt a derailed B cell response in SLE. Another possible benefit targeting the IL-21/IL-21R axis could be the expansion of T_FR_ cell differentiation and the recovery of T_FR_/T_FH_ balance. Modulating the IL-21/IL-21R signaling pathway is currently the focus of autoimmune disease treatment. At present, there is an ongoing randomized double-blind phase 1b/2 study of BOS161721 administered in SLE, but no results have been reported yet (Clinical trial nr.: NCT03371251). There is another randomized, double-blind, placebo-controlled study that aims to neutralize IL-21 cytokines using a recombinant α(IL)-21 monoclonal antibody for the treatment of rheumatoid arthritis, but so far only safety endpoints and pharmacokinetics were assessed [75]. 

**Figure 5:**
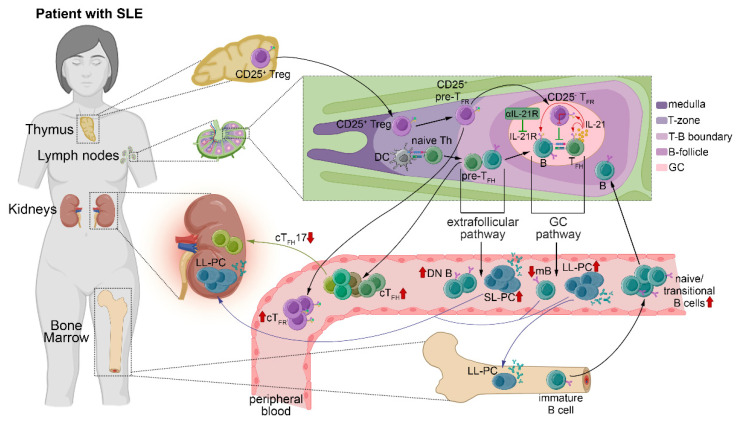
Summarizing the outcome of T_FR_/T_FH_ cell imbalance and altered B cell differentiation in SLE. Incomplete induction of self-tolerance and B cell hyperactivity induced by disturbed immune regulation are the hallmark of lupus pathogenesis. The breakdown of early B cell tolerance leads to consequential enrichment of the ratio of transitional B cells and naive/mature–naive B cells in the circulation. The microenvironment of GCs gives place to peripheral tolerance, where T_FR_ cells and T_FH_ cells collaborate to regulate the multistage differentiation of B cells. T_FH_ cells produce IL-21, which facilitates the GC reaction by promoting T_FH_ and B cell differentiation, while inhibiting T_FR_ function. The imbalance of T_FR_ cell and T_FH_ cell homeostasis in lymph nodes culminates in an increased number of activated cT_FH_ and cT_FR_ cells at the periphery, along with characteristic changes in the distribution of B cell subpopulations. We hypothesize that cT_FH_17 cells may traffic to the site of inflammation, where they collaborate with the accumulated antibody-secreting cells and memory B cells within kidney tissues, which contributes to the progression of the disease. DC, dendritic cell; DN B, double negative B cell; GC, germinal center; IL-21, interleukin-21; IL-21R, interleukin-21 receptor; LL-PC, long-lived plasma cell; mB, memory B cell; SL-PC, short-lived plasma cell; SLE, systemic lupus erythematosus; Treg, regulatory T cell; T_FR_, follicular T regulatory cell; T_FH_, follicular T helper cells. [Some icons were inspired by Biorender.com accessed on 16 August 2022.].

The strength of our study was the comprehensive analysis of the change in cT_FH_ subsets and cT_FR_ cells in SLE and its correlation with the aberrant distribution of different B cell subpopulations and relevant clinical laboratory parameters. The limitation of this study was that we used magnetically isolated CD19^+^ B cells and CD4^+^CXCR5^+^ T cells during in vitro experiments, but not sorted cT_FH_ and B cell subsets. Sorted cT_FH_ subsets without cT_FR_ cells and sorted naive B cells should be applied in future studies, to provide a more specific response in the co-culture system. Although significant advances have been made in understanding the mechanisms of T_FR_/T_FH_ balance, there are many contradictions between the previous results. Our present findings demonstrate that, besides cT_FR_/cT_FH_ imbalance, the decreased frequency of cT_FH_17 cells contributes to lupus pathogenesis, particularly lupus nephritis, and blocking the IL-21/IL-21R pathway may facilitate the development of new clinical therapeutics to restrain pathological humoral immunity in SLE (Figure 5).

## 4. Materials and Methods

### 4.1. Study Population

The study population consisted of 48 patients with SLE (47 female and 1 male; median age (25th–75th percentile): 40 (32–45) years) and 36 age and sex-matched healthy volunteers (35 female and 1 male; median age: 43 (34–47) years). All patients were recruited from the out-patient clinic for systemic autoimmune diseases at the Division of Clinical Immunology, University of Debrecen, where they received regular follow-up treatment. The mean age of disease duration was 8.9 ± 8.12 years, and the median (25th–75th percentile) disease duration was 7 (3–12) years. All SLE patients fulfilled the EULAR/American College of Rheumatology (ACR) 2019 classification criteria for lupus [76]. The disease activity was quantified by the SLEDAI (median SLEDAI: 2; range: 12), and patients were ranked according to the obtained score: inactive disease/mild activity (SLEDAI 0–4; mean: 2.06 ± 0.15, median: 2; *n* = 34) and active disease/moderate activity (SLEDAI 6–12; mean: 8.14 ± 1.99, median: 8; *n* = 14). Participants with any viral or bacterial infections, as well as patients diagnosed with other chronic or autoimmune diseases, were excluded. All the procedures were approved by the Ethics Committee of our University (protocol number: 4879-2017) and the Policy Administration Services of Public Health of the Government Office (protocol number: 1660-4/2018). Informed written consent was obtained from all the participants involved in this research, and the study was performed in agreement with the ethical standards of the Declaration of Helsinki. The serological characteristics and clinical information on the patients with lupus are detailed in Table 1.

### 4.2. Flow Cytometry Analysis 

Human PBMCs were isolated by gradient centrifugation using a Ficoll-Histopaque (Sigma-Aldrich, St Louis, MO, USA). The obtained cell suspension was stained with the following fluorochrome-conjugated monoclonal antibodies: fluorescein isothiocyanate (FITC) anti-IgD (clone: IADB6, Beckman Coulter Inc., Fullerton, CA, USA), phycoerythrin (PE) anti-CD27 (clone: 1A4CD27, Beckman Coulter), phycoerythrin-Cyanine dye 5 (PE-Cy5) anti-CD19 (clone: J3-119, Beckman Coulter), FITC anti-CD38 (clone: HIT2, BioLegend, San Diego, CA, USA), allophycocyanin (APC) anti-CD24 (clone: ML5, BioLegend), Alexa Fluor 488 anti-CXCR5 (clone: RF8B2, BD Biosciences, San Diego, CA, USA), PE anti-ICOS (clone: DX29, BD Biosciences), Peridinin-chlorophyll protein-Cyanine dye 5.5 (PerCP-Cy5.5) anti-PD-1 (clone: EH12.1, BD Biosciences), PE anti-CXCR3 (clone: G025H7, BioLegend), PerCP-Cy5.5 anti-CCR6 (clone: G034E3, BioLegend), PE anti-CD127 (clone: R34.34, Beckman Coulter), PE-Cy5 anti-CD25 (clone: B1.49.9, Beckman Coulter) and anti-CD4-APC (clone: RPA-T4, BioLegend). Cells were stained for 20 min at 4 °C in the dark, then washed twice and prepared for measurements. Multiparameter flow cytometry analysis was performed with a FACS Calibur instrument (Becton Dickinson, Franklin Lakes, NJ, USA) and further analyzed with FlowJo v10.0.7 software, (Treestar, Ashland, OR, USA). In the case of B cells, at least 5000 CD19^+^ events per sample were analyzed, while cT_FH_ cell data were collected from at least 50,000 CD4^+^ events per sample within the whole lymphocyte population. The proportions of B cell and cT_FH_ cell subsets were analyzed within the total CD19^+^ B cells and CD4^+^ T cells, respectively. Fluorescence Minus One (FMO) controls were used to identify positive populations and determine gate settings in multicolor experiments. The tSNE analyses of T_FH_ cell subsets based on immune cell flow cytometry were performed using the tSNE plugin in FlowJo v10.8.1 software. Approximate K-nearest neighbors (KNN) algorithm (ANNoy library) and fast Fourier-transform (FFT) interpolation gradient algorithm were used for data visualization [77]. CXCR5, PD-1, ICOS, CD25, CD127, CXCR3 and CCR6 parameters were selected to cluster populations with 1000 iterations and a perplexity of 30. To equalize the events for every sample, a Downsample plugin was used.

### 4.3. Cell Isolation

Circulating T_FH_ cells were isolated using a two-step procedure: at first, CD4^+^ T cells were enriched from PBMCs with a CD4^+^ T cell isolation kit; then, CXCR5^+^ T cells were purified using a CD185 (CXCR5)-biotin antibody and anti-biotin microbead (all from Miltenyi Biotec; Bergisch Gladbach, Germany) according to the manufacturer’s instructions. B cells were isolated with a CD19^+^ B cell isolation kit (Miltenyi Biotec). Cell purity was above 80% for cT_FH_-like cells and it was higher than 97% for B cells, as determined by flow cytometry (healthy controls, *n* = 3; patients with SLE, *n* = 3).

### 4.4. In Vitro cT_FH_-like Cell and B Cell Co-Culture Assay

Purified B cells were pre-activated with a BCR agonist affinity purified F(ab′)2 fragment of goat anti-human IgG + IgM (H + L) (2.5 μg/mL; Jackson ImmunoResearch, Baltimore, PA, USA) in the presence of recombinant human IL-2 and IL-10 proteins (both 50 ng/mL; PeproTech, Cranbury, NJ, USA) for 24 h at 37 °C in 5% CO_2_ milieu in Roswell Park Memorial Institute (RPMI) 1640 medium containing 10% heat-inactivated fetal bovine serum in 96-well flat-bottom plates. Activated B cells (5 × 10^4^) were co-cultured with equal numbers of autologous CD4^+^CXCR5^+^ cT_FH_ cells in 200 μL of complete RPMI medium in 96-well U-bottom plates and stimulated with SEB (1 μg/mL; Sigma-Aldrich; Steinheim, Germany). To modulate T/B cell interaction, recombinant human IL-21R Fc chimera protein (2.5 μg/mL, R&D Systems, MN, USA), as the isotype control, and recombinant human IgG1 Fc chimera protein (2.5 μg/mL, R&D Systems) was added to the culture. After 7 days, cells were harvested and stained for plasmablast phenotype using FITC-conjugated anti-CD38, PE-conjugated anti-CD27, APC-conjugated anti-CD4 monoclonal antibodies and 7-AAD viability solution. CD38^hi^CD27^+^ plasmablasts were analyzed and data acquired on a FACS Calibur flow cytometer equipped with CellQuest v3.3 software. Data were further analyzed using FlowJo v10.0.7 software. Supernatants were collected and stored at −20 °C until further measurements. 

### 4.5. ELISA to Assess B Cell Function

IgG and IgM concentrations in the supernatants were measured using enzyme-linked immunosorbent assay (ELISA) kits according to the manufacturer’s instructions (MabTech AB, Nacka Strand, Sweden).

### 4.6. Assessment of Routine Laboratory Parameters 

All patients were followed up on a routine basis, and their records contain detailed information on symptoms, clinical conditions, laboratory and other findings of each visit. Routine laboratory measurements were carried out at the Department of Laboratory Medicine, Faculty of Medicine, University of Debrecen. Blood cell counts, including total lymphocyte counts, were analyzed with the ADVIA 2120i hematology system (Siemens, Munich, Germany). Anti-dsDNA autoantibody levels (normal range: ≤20 IU/mL) were determined by ELISA with AUTOSTAT II kits (Hycor Bio-medical, Indianapolis, IN, USA), according to the manufacturer’s instructions. Serum concentrations of complement C3 (normal range: 0.9 to 1.8 g/L) and C4 (normal range: 0.1 to 0.4 g/L) proteins were measured by a quantitative turbidimetric assay (Dialab GmbH, Wiener Neudorf, Austria). ICs were detected by the polyethylene glycol precipitation method. The laboratory reference range was an extinction of 0 to 170 for IC. The results were given according to the analytical measurement range stated in the manufacturers’ instructions. 

### 4.7. Statistical Analysis 

Data and statistical analyses and graphic representation were carried out with GraphPad Prism v8 software (GraphPad Software, San Diego, USA). Descriptive data were represented in box plots of inter-quartile range (IQR) with a line in the middle as the median value. Tukey whiskers show the 25th percentile minus 1.5 times IQR and the 75th percentile plus 1.5 times IQR. Functional assay data displayed in bar charts with mean ± standard deviation (SD). Kolmogorov-Smirnov and Shapiro–Wilk normality tests were used to assess normal distribution. The significance for the differences between two groups was determined by the Mann-Whitney test. In the case of multiple comparisons, two-way analysis of variance (ANOVA) with Sidak or Tukey’s post hoc test was used. Statistics and visualization of the correlation matrices were carried out with R Version 4.1.1 and RStudio Version 1.4.1717 [78,79], using the Hmisc [80] and corrplot [81] packages. Correlations between the percentages of cell subsets and routine laboratory parameters were analyzed by Spearman’s rank correlation test. Differences were considered statistically significant at *p* ≤ 0.05.

## Figures and Tables

**Table 1:** Demographic characteristic of patients with SLE and healthy controls.

Characteristics	SLE (*n* = 48)	HC (*n* = 36)	*p* Value
Demographics			
Age, median years	40 (32–45)	43 (34–47)	0.3121
Gender, M/F	1/47	1/36	-
SLEDAI, mean ± SD	3.83 ± 3.14	NA	-
SLEDAI, median	2 (2–6)	NA	-
Laboratory variables			
Lymphocytes, 10^9^/L	1.17 (0.75–1.71)	1.71 (1.39–2.20)	0.0004
Anti-dsDNA titer, IU/mL	28.7 (21.3–68.1)	NA	-
Anti-dsDNA, n (%)	36 (75.00)	NA	-
IC, titer, extinction	72 (61–109)	NA	-
C3, titer, g/L	0.96 (0.75–1.12)	NA	-
C3, n (%)	19 (39.58)	NA	-
C4, titer, g/L	0.13 (0.08–0.16)	NA	-
C4, n (%)	15 (31.25)	NA	-
Organ involvement			
Renal, n (%)	15 (31.25)	NA	-
Cutaneous, n (%)	13 (27.08)	NA	-
Medications at time of study			
No therapy, n (%)	6 (12.50)	NA	-
Methylprednisolone, n (%)	27 (56.25)	NA	-
Methylprednisolone, median, mg/day	4 (4–4)	NA	-
Chloroquine, n (%)	27 (56.25)	NA	-
Hydroxychloroquine, n (%)	3 (6.25)	NA	-
Azathioprine, n (%)	8 (16.67)	NA	-

For continuous variables, data are displayed as mean ± SD or median (25th–75th percentile). For categorical variables, data are shown as count (percentage). NA, no data available; SLE, systemic lupus erythematosus; SLEDAI, SLE Disease Activity Index; HC, healthy control; IC, immune complex; C, complement component.

## Data Availability

The data presented in this study are available in the article’s Figures and Tables, as well as in Supplementary Materials.

## Supplementary Materials

The following supporting information can be downloaded at: https://www.mdpi.com/article/10.3390/ijms232012209/s1. Supplemetary Figure S1: Peripheral blood T_FH_ cells represent a circulating pool of CD45RA^−^CD4^+^ memory T cells. Supplemetary Figure S2: The distribution of cT_FH_ subsets in patients with SLE and their association with disease activity and the presence of autoantibody. Supplemetary Figure S3: The distribution of activated cT_FH_ cells, cT_FH_ subsets, cT_FR_ cells and B cell subsets in SLE patients and healthy controls subjected to IL-21R inhibition analysis. Supplementary Table S1: Routine laboratory and clinical characteristic of patients with SLE subjected to IL-21R inhibition analysis.

## References

[1] Thanou A., Jupe E., Purushothaman M., Niewold T.B., Munroe M.E. (2021). Clinical disease activity and flare in SLE: Current concepts and novel biomarkers. J. Autoimmun..

[2] Tarr T., Papp G., Nagy N., Cserep E., Zeher M. (2017). Chronic high-dose glucocorticoid therapy triggers the development of chronic organ damage and worsens disease outcome in systemic lupus erythematosus. Clin. Rheumatol..

[3] Kaul A., Gordon C., Crow M.K., Touma Z., Urowitz M.B., van Vollenhoven R., Ruiz-Irastorza G., Hughes G. (2016). Systemic lupus erythematosus. Nat. Rev. Dis. Primers.

[4] Li Q., Wu H., Liao W., Zhao M., Chan V., Li L., Zheng M., Chen G., Zhang J., Lau C.S. (2018). A comprehensive review of immune-mediated dermatopathology in systemic lupus erythematosus. J. Autoimmun..

[5] Anders H.J., Saxena R., Zhao M.H., Parodis I., Salmon J.E., Mohan C. (2020). Lupus nephritis. Nat. Rev. Dis. Primers.

[6] Cashman K.S., Jenks S.A., Woodruff M.C., Tomar D., Tipton C.M., Scharer C.D., Eun-Hyung Lee F., Boss J.M., Sanz I. (2019). Understanding and measuring human B-cell tolerance and its breakdown in autoimmune disease. Immunol. Rev..

[7] Hamilton J.A., Hsu H.C., Mountz J.D. (2019). Autoreactive B cells in SLE, villains or innocent bystanders?. Immunol. Rev..

[8] Yurasov S., Wardemann H., Hammersen J., Tsuiji M., Meffre E., Pascual V., Nussenzweig M.C. (2005). Defective B cell tolerance checkpoints in systemic lupus erythematosus. J. Exp. Med..

[9] Ding T., Su R., Wu R., Xue H., Wang Y., Su R., Gao C., Li X., Wang C. (2021). Frontiers of Autoantibodies in Autoimmune Disorders: Crosstalk Between Tfh/Tfr and Regulatory B Cells. Front. Immunol..

[10] Chang A., Henderson S.G., Brandt D., Liu N., Guttikonda R., Hsieh C., Kaverina N., Utset T.O., Meehan S.M., Quigg R.J. (2011). In situ B cell-mediated immune responses and tubulointerstitial inflammation in human lupus nephritis. J. Immunol..

[11] Dorraji S.E., Kanapathippillai P., Hovd A.K., Stenersrod M.R., Horvei K.D., Ursvik A., Figenschau S.L., Thiyagarajan D., Fenton C.G., Pedersen H.L. (2020). Kidney Tertiary Lymphoid Structures in Lupus Nephritis Develop into Large Interconnected Networks and Resemble Lymph Nodes in Gene Signature. Am. J. Pathol..

[12] Kang S., Fedoriw Y., Brenneman E.K., Truong Y.K., Kikly K., Vilen B.J. (2017). BAFF Induces Tertiary Lymphoid Structures and Positions T Cells within the Glomeruli during Lupus Nephritis. J. Immunol..

[13] Corsiero E., Nerviani A., Bombardieri M., Pitzalis C. (2016). Ectopic Lymphoid Structures: Powerhouse of Autoimmunity. Front. Immunol..

[14] Crotty S. (2019). T Follicular Helper Cell Biology: A Decade of Discovery and Diseases. Immunity.

[15] Kim C.H., Rott L.S., Clark-Lewis I., Campbell D.J., Wu L., Butcher E.C. (2001). Subspecialization of CXCR5^+^ T cells: B helper activity is focused in a germinal center-localized subset of CXCR5^+^ T cells. J. Exp. Med..

[16] Hardtke S., Ohl L., Forster R. (2005). Balanced expression of CXCR5 and CCR7 on follicular T helper cells determines their transient positioning to lymph node follicles and is essential for efficient B-cell help. Blood.

[17] Nurieva R.I., Chung Y., Martinez G.J., Yang X.O., Tanaka S., Matskevitch T.D., Wang Y.H., Dong C. (2009). Bcl6 mediates the development of T follicular helper cells. Science.

[18] Qi H. (2016). T follicular helper cells in space-time. Nat. Rev. Immunol..

[19] Vogelzang A., McGuire H.M., Yu D., Sprent J., Mackay C.R., King C. (2008). A fundamental role for interleukin-21 in the generation of T follicular helper cells. Immunity.

[20] Bryant V.L., Ma C.S., Avery D.T., Li Y., Good K.L., Corcoran L.M., de Waal Malefyt R., Tangye S.G. (2007). Cytokine-mediated regulation of human B cell differentiation into Ig-secreting cells: Predominant role of IL-21 produced by CXCR5+ T follicular helper cells. J. Immunol..

[21] Linterman M.A., Pierson W., Lee S.K., Kallies A., Kawamoto S., Rayner T.F., Srivastava M., Divekar D.P., Beaton L., Hogan J.J. (2011). Foxp3^+^ follicular regulatory T cells control the germinal center response. Nat. Med..

[22] Fonseca V.R., Ribeiro F., Graca L. (2019). T follicular regulatory (Tfr) cells: Dissecting the complexity of Tfr-cell compartments. Immunol. Rev..

[23] Deng J., Wei Y., Fonseca V.R., Graca L., Yu D. (2019). T follicular helper cells and T follicular regulatory cells in rheumatic diseases. Nat. Rev. Rheumatol..

[24] Vella L.A., Buggert M., Manne S., Herati R.S., Sayin I., Kuri-Cervantes L., Bukh Brody I., O’Boyle K.C., Kaprielian H., Giles J.R. (2019). T follicular helper cells in human efferent lymph retain lymphoid characteristics. J. Clin. Investig..

[25] Morita R., Schmitt N., Bentebibel S.E., Ranganathan R., Bourdery L., Zurawski G., Foucat E., Dullaers M., Oh S., Sabzghabaei N. (2011). Human blood CXCR5(^+^)CD4(^+^) T cells are counterparts of T follicular cells and contain specific subsets that differentially support antibody secretion. Immunity.

[26] He J., Tsai L.M., Leong Y.A., Hu X., Ma C.S., Chevalier N., Sun X., Vandenberg K., Rockman S., Ding Y. (2013). Circulating precursor CCR7(lo)PD-1(hi) CXCR5(^+^) CD4(^+^) T cells indicate Tfh cell activity and promote antibody responses upon antigen reexposure. Immunity.

[27] Ueno H. (2016). T follicular helper cells in human autoimmunity. Curr. Opin. Immunol..

[28] Bentebibel S.E., Lopez S., Obermoser G., Schmitt N., Mueller C., Harrod C., Flano E., Mejias A., Albrecht R.A., Blankenship D. (2013). Induction of ICOS+CXCR3+CXCR5+ TH cells correlates with antibody responses to influenza vaccination. Sci. Transl. Med..

[29] Fonseca V.R., Agua-Doce A., Maceiras A.R., Pierson W., Ribeiro F., Romao V.C., Pires A.R., da Silva S.L., Fonseca J.E., Sousa A.E. (2017). Human blood T_fr_ cells are indicators of ongoing humoral activity not fully licensed with suppressive function. Sci. Immunol..

[30] Wing J.B., Kitagawa Y., Locci M., Hume H., Tay C., Morita T., Kidani Y., Matsuda K., Inoue T., Kurosaki T. (2017). A distinct subpopulation of CD25(^−^) T-follicular regulatory cells localizes in the germinal centers. Proc. Natl. Acad. Sci. USA.

[31] Simpson N., Gatenby P.A., Wilson A., Malik S., Fulcher D.A., Tangye S.G., Manku H., Vyse T.J., Roncador G., Huttley G.A. (2010). Expansion of circulating T cells resembling follicular helper T cells is a fixed phenotype that identifies a subset of severe systemic lupus erythematosus. Arthritis Rheum. Off. J. Am. Coll. Rheumatol..

[32] Choi J.Y., Ho J.H., Pasoto S.G., Bunin V., Kim S.T., Carrasco S., Borba E.F., Goncalves C.R., Costa P.R., Kallas E.G. (2015). Circulating follicular helper-like T cells in systemic lupus erythematosus: Association with disease activity. Arthritis Rheumatol..

[33] Le Coz C., Joublin A., Pasquali J.L., Korganow A.S., Dumortier H., Monneaux F. (2013). Circulating TFH subset distribution is strongly affected in lupus patients with an active disease. PLoS ONE.

[34] Xu B., Wang S., Zhou M., Huang Y., Fu R., Guo C., Chen J., Zhao J., Gaskin F., Fu S.M. (2017). The ratio of circulating follicular T helper cell to follicular T regulatory cell is correlated with disease activity in systemic lupus erythematosus. Clin. Immunol..

[35] Liu C., Wang D., Song Y., Lu S., Zhao J., Wang H. (2018). Increased circulating CD4(^+^)CXCR5(^+^)FoxP3(^+^) follicular regulatory T cells correlated with severity of systemic lupus erythematosus patients. Int. Immunopharmacol..

[36] Stebegg M., Kumar S.D., Silva-Cayetano A., Fonseca V.R., Linterman M.A., Graca L. (2018). Regulation of the Germinal Center Response. Front. Immunol..

[37] Weber J.P., Fuhrmann F., Hutloff A. (2012). T-follicular helper cells survive as long-term memory cells. Eur. J. Immunol..

[38] Sage P.T., Alvarez D., Godec J., von Andrian U.H., Sharpe A.H. (2014). Circulating T follicular regulatory and helper cells have memory-like properties. J. Clin. Investig..

[39] Zhang X., Lindwall E., Gauthier C., Lyman J., Spencer N., Alarakhia A., Fraser A., Ing S., Chen M., Webb-Detiege T. (2015). Circulating CXCR5^+^CD4^+^helper T cells in systemic lupus erythematosus patients share phenotypic properties with germinal center follicular helper T cells and promote antibody production. Lupus.

[40] Yang X., Yang J., Chu Y., Xue Y., Xuan D., Zheng S., Zou H. (2014). T follicular helper cells and regulatory B cells dynamics in systemic lupus erythematosus. PLoS ONE.

[41] Feng X., Wang D., Chen J., Lu L., Hua B., Li X., Tsao B.P., Sun L. (2012). Inhibition of aberrant circulating Tfh cell proportions by corticosteroids in patients with systemic lupus erythematosus. PLoS ONE.

[42] Xu H., Liu J., Cui X., Zuo Y., Zhang Z., Li Y., Tao R., Li Y., Pang J. (2015). Increased frequency of circulating follicular helper T cells in lupus patients is associated with autoantibody production in a CD40L-dependent manner. Cell Immunol..

[43] Zhou H., Hu B., Huang N., Mo X., Li W., Zhang B., Wei B., Gao M., Wang Y., Liu X. (2018). Aberrant T cell subsets and cytokines expression profile in systemic lupus erythematosus. Clin. Rheumatol..

[44] Szabo K., Papp G., Szanto A., Tarr T., Zeher M. (2016). A comprehensive investigation on the distribution of circulating follicular T helper cells and B cell subsets in primary Sjogren’s syndrome and systemic lupus erythematosus. Clin. Exp. Immunol..

[45] Sasaki T., Bracero S., Keegan J., Chen L., Cao Y., Stevens E., Qu Y., Wang G., Nguyen J., Sparks J.A. (2022). Longitudinal immune cell profiling in early systemic lupus erythematosus. Arthritis Rheumatol..

[46] Turner J.E., Paust H.J., Steinmetz O.M., Peters A., Riedel J.H., Erhardt A., Wegscheid C., Velden J., Fehr S., Mittrucker H.W. (2010). CCR6 recruits regulatory T cells and Th17 cells to the kidney in glomerulonephritis. J. Am. Soc. Nephrol..

[47] Hao H., Nakayamada S., Yamagata K., Ohkubo N., Iwata S., Inoue Y., Zhang M., Zhang T., Kanda Satoh Y., Shan Y. (2021). Conversion of T Follicular Helper Cells to T Follicular Regulatory Cells by Interleukin-2 Through Transcriptional Regulation in Systemic Lupus Erythematosus. Arthritis Rheumatol..

[48] Kurata I., Mikami N., Ohyama A., Osada A., Kondo Y., Tsuboi H., Sumida T., Matsumoto I. (2021). Impaired function of PD-1(^+^) follicular regulatory T cells in systemic lupus erythematosus. Clin. Exp. Immunol..

[49] Liu W., Putnam A.L., Xu-Yu Z., Szot G.L., Lee M.R., Zhu S., Gottlieb P.A., Kapranov P., Gingeras T.R., Fazekas de St Groth B. (2006). CD127 expression inversely correlates with FoxP3 and suppressive function of human CD4^+^ T reg cells. J. Exp. Med..

[50] Liang K., He J., Wei Y., Zeng Q., Gong D., Qin J., Ding H., Chen Z., Zhou P., Niu P. (2021). Sustained low-dose interleukin-2 therapy alleviates pathogenic humoral immunity via elevating the Tfr/Tfh ratio in lupus. Clin. Transl. Immunol..

[51] Miao M., Xiao X., Tian J., Zhufeng Y., Feng R., Zhang R., Chen J., Zhang X., Huang B., Jin Y. (2021). Therapeutic potential of targeting Tfr/Tfh cell balance by low-dose-IL-2 in active SLE: A post hoc analysis from a double-blind RCT study. Arthritis Res. Ther..

[52] Mao M., Xu S., Lin L., Dong D., Xue M., He S., Cai G. (2022). Impact of Corticosteroids on the Proportions of Circulating T_fh_ Cell Subsets in Patients With Systemic Lupus Erythematous. Front. Med..

[53] Yang M., Cao P., Zhao Z., Wang Z., Jia C., Guo Y., Yin H., Zhao M., Ding Y., Wu H. (2021). An Enhanced Expression Level of CXCR3 on Tfh-like Cells from Lupus Skin Lesions Rather Than Lupus Peripheral Blood. Clin. Immunol..

[54] Paquissi F.C., Abensur H. (2021). The Th17/IL-17 Axis and Kidney Diseases, With Focus on Lupus Nephritis. Front. Med..

[55] Riedel J.H., Turner J.E., Panzer U. (2021). T helper cell trafficking in autoimmune kidney diseases. Cell Tissue Res..

[56] Wu X., Guo J., Ding R., Lv B., Bi L. (2015). CXCL13 blockade attenuates lupus nephritis of MRL/lpr mice. Acta Histochem..

[57] Abraham R., Durkee M.S., Ai J., Veselits M., Casella G., Asano Y., Chang A., Ko K., Oshinsky C., Peninger E. (2022). Specific in situ inflammatory states associate with progression to renal failure in lupus nephritis. J. Clin. Investig..

[58] Liarski V.M., Kaverina N., Chang A., Brandt D., Yanez D., Talasnik L., Carlesso G., Herbst R., Utset T.O., Labno C. (2014). Cell distance mapping identifies functional T follicular helper cells in inflamed human renal tissue. Sci. Transl. Med..

[59] Dunlap G.S., Billi A.C., Xing X., Ma F., Maz M.P., Tsoi L.C., Wasikowski R., Hodgin J.B., Gudjonsson J.E., Kahlenberg J.M. (2022). Single-cell transcriptomics reveals distinct effector profiles of infiltrating T cells in lupus skin and kidney. JCI Insight.

[60] Koga T., Otomo K., Mizui M., Yoshida N., Umeda M., Ichinose K., Kawakami A., Tsokos G.C. (2016). Calcium/Calmodulin-Dependent Kinase IV Facilitates the Recruitment of Interleukin-17-Producing Cells to Target Organs Through the CCR6/CCL20 Axis in Th17 Cell-Driven Inflammatory Diseases. Arthritis Rheumatol..

[61] Tipton C.M., Fucile C.F., Darce J., Chida A., Ichikawa T., Gregoretti I., Schieferl S., Hom J., Jenks S., Feldman R.J. (2015). Diversity, cellular origin and autoreactivity of antibody-secreting cell population expansions in acute systemic lupus erythematosus. Nat. Immunol..

[62] Rodriguez-Bayona B., Ramos-Amaya A., Perez-Venegas J.J., Rodriguez C., Brieva J.A. (2010). Decreased frequency and activated phenotype of blood CD27 IgD IgM B lymphocytes is a permanent abnormality in systemic lupus erythematosus patients. Arthritis Res. Ther..

[63] Dorner T., Lipsky P.E. (2004). Correlation of circulating CD27high plasma cells and disease activity in systemic lupus erythematosus. Lupus.

[64] Sanz I., Wei C., Jenks S.A., Cashman K.S., Tipton C., Woodruff M.C., Hom J., Lee F.E. (2019). Challenges and Opportunities for Consistent Classification of Human B Cell and Plasma Cell Populations. Front. Immunol..

[65] Horisberger A., Humbel M., Fluder N., Bellanger F., Fenwick C., Ribi C., Comte D. (2022). Measurement of circulating CD21(^−^)CD27(^−^) B lymphocytes in SLE patients is associated with disease activity independently of conventional serological biomarkers. Sci. Rep..

[66] Wei C., Anolik J., Cappione A., Zheng B., Pugh-Bernard A., Brooks J., Lee E.H., Milner E.C., Sanz I. (2007). A new population of cells lacking expression of CD27 represents a notable component of the B cell memory compartment in systemic lupus erythematosus. J. Immunol..

[67] Wang S., Wang J., Kumar V., Karnell J.L., Naiman B., Gross P.S., Rahman S., Zerrouki K., Hanna R., Morehouse C. (2018). IL-21 drives expansion and plasma cell differentiation of autoreactive CD11c(hi)T-bet(^+^) B cells in SLE. Nat. Commun..

[68] Ding Y., Li J., Yang P., Luo B., Wu Q., Zajac A.J., Wildner O., Hsu H.C., Mountz J.D. (2014). Interleukin-21 promotes germinal center reaction by skewing the follicular regulatory T cell to follicular helper T cell balance in autoimmune BXD2 mice. Arthritis Rheumatol..

[69] Jandl C., Liu S.M., Canete P.F., Warren J., Hughes W.E., Vogelzang A., Webster K., Craig M.E., Uzel G., Dent A. (2017). IL-21 restricts T follicular regulatory T cell proliferation through Bcl-6 mediated inhibition of responsiveness to IL-2. Nat. Commun..

[70] Kim V., Lee K., Tian H., Jang S.H., Diamond B., Kim S.J. (2022). IL-17-producing follicular Th cells enhance plasma cell differentiation in lupus-prone mice. JCI Insight.

[71] Herber D., Brown T.P., Liang S., Young D.A., Collins M., Dunussi-Joannopoulos K. (2007). IL-21 has a pathogenic role in a lupus-prone mouse model and its blockade with IL-21R.Fc reduces disease progression. J. Immunol..

[72] Wu H., Deng Y., Long D., Yang M., Li Q., Feng Y., Chen Y., Qiu H., Huang X., He Z. (2022). The IL-21-TET2-AIM2-c-MAF pathway drives the T follicular helper cell response in lupus-like disease. Clin. Transl. Med..

[73] Choi J.Y., Seth A., Kashgarian M., Terrillon S., Fung E., Huang L., Wang L.C., Craft J. (2017). Disruption of Pathogenic Cellular Networks by IL-21 Blockade Leads to Disease Amelioration in Murine Lupus. J. Immunol..

[74] Terrier B., Costedoat-Chalumeau N., Garrido M., Geri G., Rosenzwajg M., Musset L., Klatzmann D., Saadoun D., Cacoub P. (2012). Interleukin 21 correlates with T cell and B cell subset alterations in systemic lupus erythematosus. J. Rheumatol..

[75] Ignatenko S., Skrumsager B.K., Mouritzen U. (2016). Safety, PK, and PD of recombinant anti-interleukin-21 monoclonal antibody in a first-in-human trial. Int. J. Clin. Pharmacol. Ther..

[76] Petri M., Orbai A.M., Alarcon G.S., Gordon C., Merrill J.T., Fortin P.R., Bruce I.N., Isenberg D., Wallace D.J., Nived O. (2012). Derivation and validation of the Systemic Lupus International Collaborating Clinics classification criteria for systemic lupus erythematosus. Arthritis Rheum..

[77] Linderman G.C., Rachh M., Hoskins J.G., Steinerberger S., Kluger Y. (2019). Fast interpolation-based t-SNE for improved visualization of single-cell RNA-seq data. Nat. Methods.

[78] RStudio Team RStudio: Integrated Development for R. http://www.rstudio.com/.

[79] R Core Team R: A Language and Environment for Statistical Computing. https://www.R-project.org/.

[80] Harrell F.E., Dupont C. (2022). R Package.

[81] Wei T., Simko V. (2021). R Package.

