# Supplementary material for: Altered Circulating Follicular T Helper Cell Subsets and Follicular T Regulatory Cells Are Indicators of a Derailed B Cell Response in Lupus, Which Could Be Modified by Targeting IL-21R
Source: Int J Mol Sci. 2022 Oct 13;23(20):12209. doi: 10.3390/ijms232012209 (PMC9602506; doi:10.3390/ijms232012209)
Supplement: Supplementary file 1 [file ijms-23-12209-s001.zip › ijms-1906263-supplementary.pdf]

International Journal of *Molecular Sciences*

MDPI

Supplementary Material

Altered circulating follicular helper T cell subsets and follicular regulatory T cells are indicators of a derailed B cell response in lupus, which could be modified by targeting IL-21R

Krisztina Szabó [1,*], Ilona Jámbor [1], Kitti Pázmándi [2], Nikolett Nagy [1], Gábor Papp [1†] and Tünde Tarr [1†]

1 Division of Clinical Immunology, Institute of Internal Medicine, Faculty of Medicine, University of Debrecen, H-4032 Debrecen, Hungary
2 Department of Immunology, Faculty of Medicine, University of Debrecen, Debrecen, Hungary
* Correspondence: krisztinaszabo@med.unideb.hu
† These authors share last authorship.

Supplemetary Figure S1.

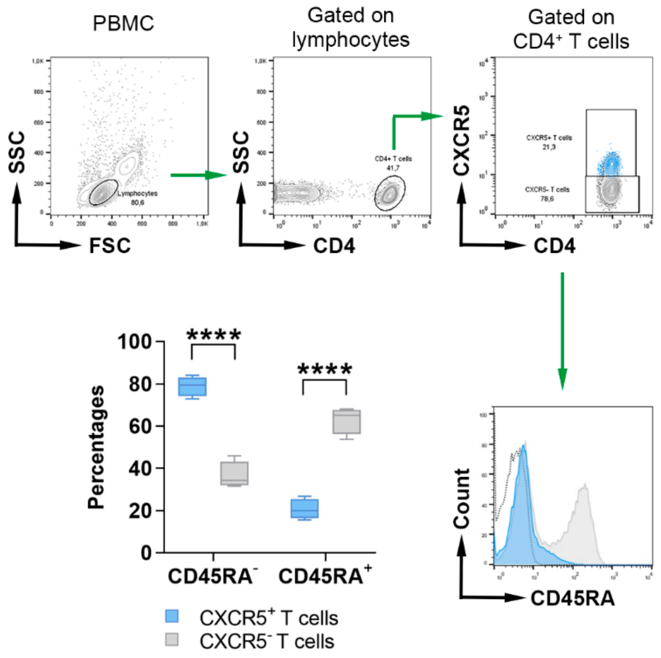

Supplemetary Figure S1. Peripheral blood TFH cells represent a circulating pool of CD45RA⁻CD4⁺ memory T cells. The representative contour plots show the gating strategy of CD4⁺CXCR5⁺ T cells. A representative histogram demonstrates the expression of CD45RA on CD4⁺CXCR5⁻ T cells (clear grey) and CD4⁺CXCR5⁺ T cells (blue shaded). CD45RA-PE Fluorescence Minus One (FMO) controls showed with dotted line. Percentages of CD45RA⁻ and CD45RA⁺ cells in CXCR5⁻ and CXCR5⁺ T cells in healthy individuals (n = 4). Two-way analysis of variance (ANOVA) with Sidak post hoc test was used. Box plots represent the interquartile range (IQR) with a line in the middle as median. Statistically significant differences are indicated by ****$p < 0.0001$.

Supplementary Figure S2.

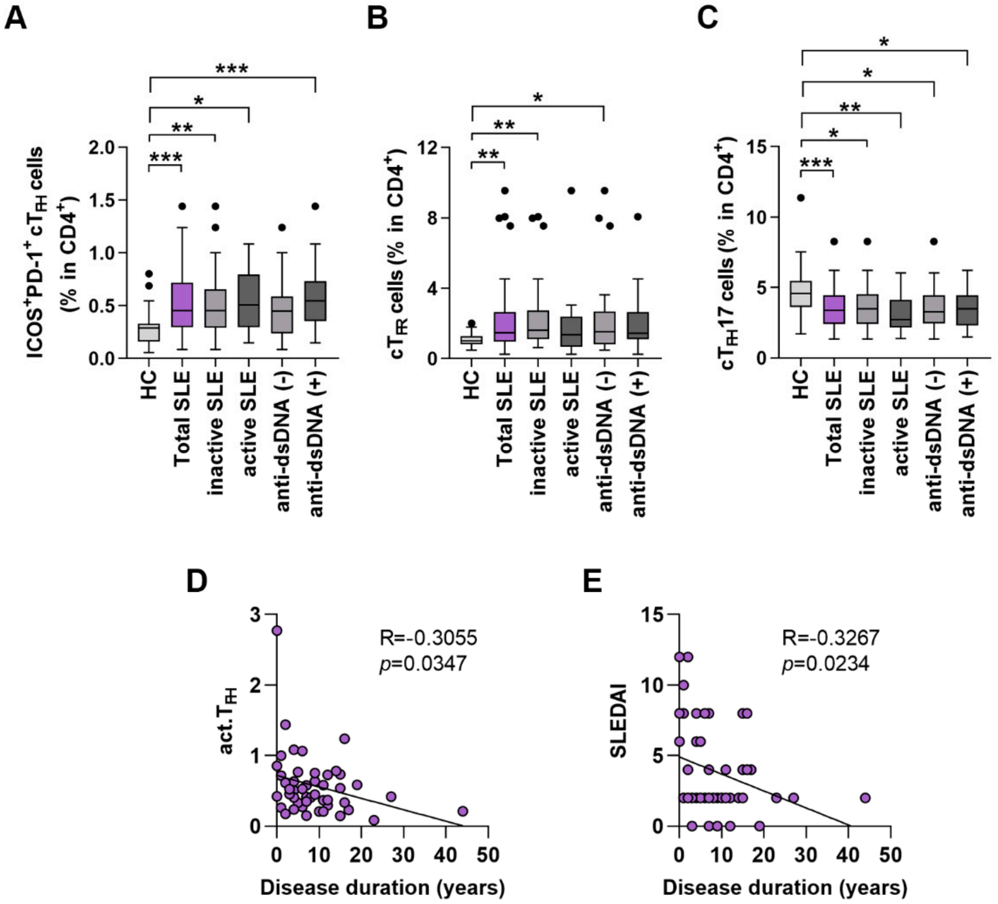

Supplemetary Figure S2. The distribution of cT_{FH} subsets in patients with SLE and their association with disease activity and the presence of autoantibody. PBMCs were isolated from 48 SLE patients and 36 healthy controls (HC) then were stained with fluorochrome-conjugated monoclonal antibodies as described previously. The percentages of blood cT_{FH} cell subsets were quantified as their percentage within $CD4^+$ lymphocytes. **(A)** Percentages of $ICOS^+PD\text{-}1^+$ activated cT_{FH} subsets in lupus patients with inactive/mild (SLEDAI 0-4, n = 34) and active/moderate (SLEDAI 6-12; n = 14) activity, SLE with anti-dsDNA (30 < IU/mL; n = 24) and without anti-dsDNA (0-30 IU/mL; n = 24) and HCs. **(B)** Proportions of $CD25^+CD127^{lo/-}$ cT_{FR} cells in SLE patients with inactive/mild (SLEDAI 0-4, n = 34) and active/moderate (SLEDAI 6-12; n = 14) activity, SLE with anti-dsDNA (30 < IU/mL; n = 24) and without anti-dsDNA (0-30 IU/mL; n = 24) and HCs. **(C)** Frequencies of $CXCR3^-CCR6^+$ $cT_{FH}17$ cells in SLE patients with inactive/mild (SLEDAI 0-4, n = 34) and active/moderate (SLEDAI 6-12; n = 14) activity, SLE with anti-dsDNA (30 < IU/mL; n = 24) and without anti-dsDNA (0-30 IU/mL; n = 24) and HCs. **(D)** Correlation analysis between the duration of the disease and the percentages of activated cT_{FH} cells. **(E)** Correlation analysis between the duration of the disease and SLEDAI scores. Kruskal–Wallis test with Dunn's multiple comparisons test was used. Correlation analysis was performed using Spearman's test. Box plots represent the interquartile range (IQR) with a line in the middle as median. Statistically significant differences are indicated by *$p < 0.05$; **$p < 0.01$; ***$p < 0.001$.

Supplementary Figure S3.

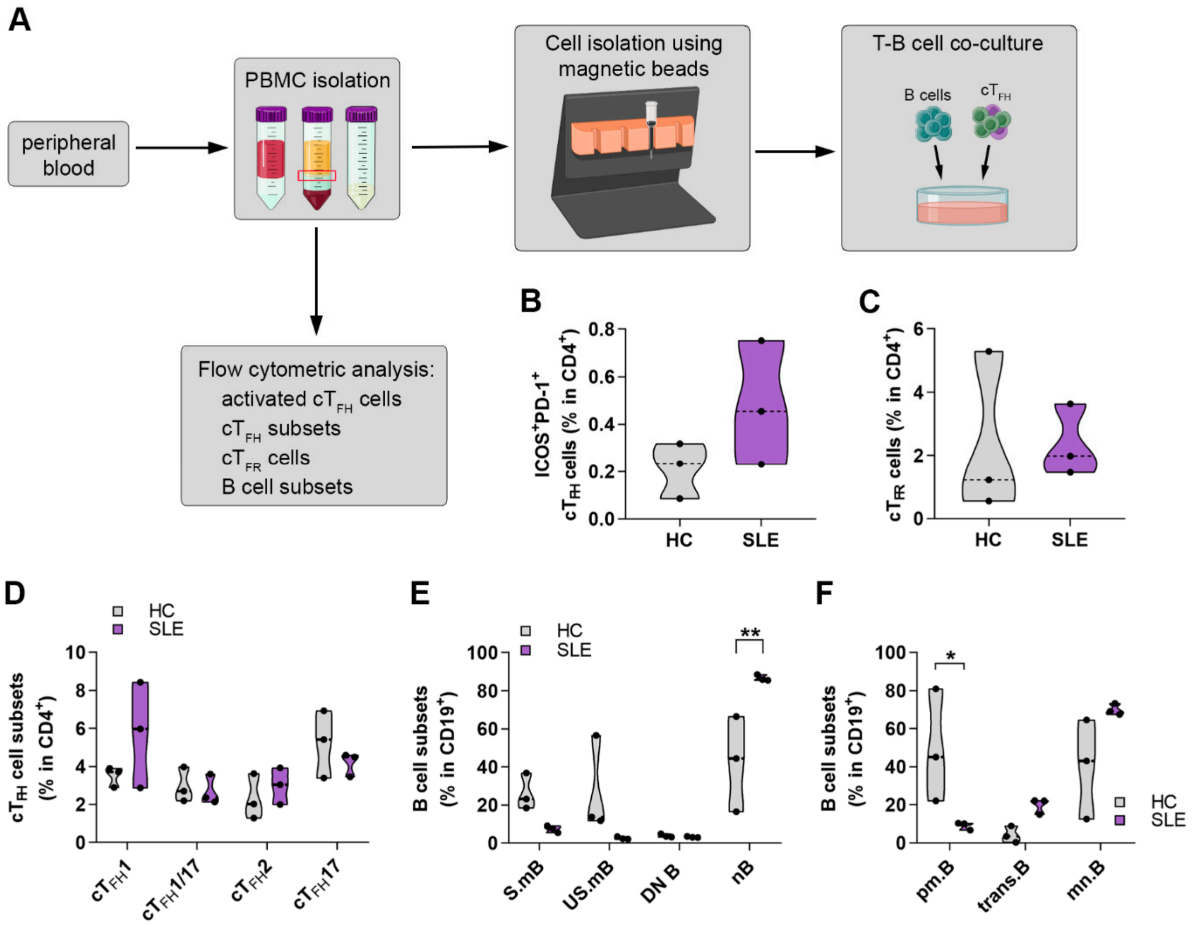

Supplemetary Figure S3. The distribution of activated cTFH cells, cTFH subsets, cTFR cells and B cell subsets in SLE patients and healthy controls subjected to IL-21R inhibition analysis. **(A)** Schematic representation of the workflow. Before proceeding to magnetic isolation, PBMCs from patients (n = 3) and healthy controls (HC, n = 3) were stained with fluorochrome-conjugated monoclonal antibodies as described in the main text. The percentages of blood cTFH cell subsets were quantified within $CD4^+$ lymphocytes while the frequencies of B cell subsets were quantified within $CD19^+$ B cells. **(B)** Percentages of $ICOS^+PD\text{-}1^+$ activated cTFH cells. **(C)** Proportions of $CD25^+CD127^{lo/-}$ cTFR cells. **(D)** Frequencies of cTFH subsets. **(E)** Proportions of naïve and memory B cell subsets: IgD^+CD27^- naive B (nB), IgD^+CD27^+ un-switched memory B (US.mB), IgD^-CD27^+ switched memory B cell (S.mB), IgD^-CD27^- double negative (DN) B cells. **(F)** Percentages of trans.B, mn.B and pm.B cell subpopulations: $CD38^{hi}CD24^{hi}CD27^-$ transitional B (trans.B), $CD38^{int}CD24^{int}$ mature-naive B (mn.B), $CD38^-CD24^{hi}CD27^+$ primarily memory B (pm.B) cells. Unpaired t-test, Mann-Whitney test or two-way analysis of variance (ANOVA) with Sidak post hoc test was used. Violin plots with dashed line in the middle as median. Statistically significant differences are indicated by $*p < 0.05$; $**p < 0.01$. [Magnetic isolation panel was created by Biorender.com.]

Supplementary Table S1.

Supplementary Table S1. Routine laboratory and clinical characteristic of patients with SLE subjected to IL-21R inhibition analysis.

No.	Anti-dsDNA titer, IU/mL	IC titer, extinction	C3 titer, g/L	C4 titer, g/L	Clinical manifestations
1	169.3	76.0	0.51	0.09	malar rash, photosensitivity
2	24.3	138.0	0.94	0.10	non-erosive polyarthritis, Raynaud's phenomenon
3	15.9	61.0	1.01	0.14	malar rash, photosensitivity

SLE, systemic lupus erythematosus; IC, immune complex; C, complement component.
